# Design and Methods of a Synchronous Online Motivational Interviewing Intervention for Weight Management

**DOI:** 10.2196/resprot.5382

**Published:** 2016-04-19

**Authors:** Rebecca Anne Krukowski, Vicki DiLillo, Krista Ingle, Jean Ruth Harvey, Delia Smith West

**Affiliations:** ^1^ Center for Population Sciences Department of Preventive Medicine University of Tennessee Health Science Center Memphis, TN United States; ^2^ Ohio Wesleyan University Department of Psychology Delaware, OH United States; ^3^ Department of Psychiatry and Behavioral Sciences Duke University Medical Center Durham, NC United States; ^4^ Department of Nutrition and Food Sciences University of Vermont Burlington, VT United States; ^5^ Department of Exercise Science Arnold School of Public Health University of South Carolina Columbia, SC United States

**Keywords:** technology, weight loss, motivational interviewing, methodology

## Abstract

**Background:**

While Internet-based weight management programs can facilitate access to and engagement in evidence-based lifestyle weight loss programs, the results have generally not been as effective as in-person programs. Furthermore, motivational interviewing (MI) has shown promise as a technique for enhancing weight loss outcomes within face-to-face programs.

**Objective:**

This paper describes the design, intervention development, and analysis of a therapist-delivered online MI intervention for weight loss in the context of an online weight loss program.

**Methods:**

The MI intervention is delivered within the context of a randomized controlled trial examining the efficacy of an 18-month, group-based, online behavioral weight control program plus individually administered, synchronous online MI sessions relative to the group-based program alone. Six individual 30-minute MI sessions are conducted in private chat rooms over 18 months by doctoral-level psychologists. Sessions use a semistructured interview format for content and session flow and incorporate core MI components (eg, collaborative agenda setting, open-ended questions, reflective listening and summary statements, objective data, and a focus on evoking and amplifying change talk).

**Results:**

The project was funded in 2010 and enrollment was completed in 2012. Data analysis is currently under way and the first results are expected in 2016.

**Conclusions:**

This is the first trial to test the efficacy of a synchronous online, one-on-one MI intervention designed to augment an online group behavioral weight loss program. If the addition of MI sessions proves to be successful, this intervention could be disseminated to enhance other distance-based weight loss interventions.

**Trial Registration:**

Clinicaltrials.gov NCT01232699; https://clinicaltrials.gov/ct2/show/NCT01232699

## Introduction

Obesity is one of the most pressing public health problems currently facing the United States, with about two-thirds of the adult population categorized as overweight or obese [ 1]. Fortunately, evidence-based lifestyle weight control programs have been shown to produce clinically significant weight losses, with studies reporting average weight losses of 7%-9% [ 2, 3], a magnitude of weight reduction that confers substantial health benefits [ 4]. However, access to and engagement in these beneficial programs may be challenging for some individuals because they lack geographic proximity to evidence-based weight management programs and/or they have time constraints associated with travel to the treatment center. Internet-based weight management programs have emerged as a method that may reduce some of these barriers [ 5- 11]. Indeed, the superior incremental cost-effectiveness of one Internet-delivered behavioral weight control program reflected savings in participant travel time [ 12].

However, while the weight losses achieved in synchronous online interventions are quite promising (approximately 5% of baseline weight), they are not as substantial as those achieved with the identical program delivered in-person (approximately 8% of baseline weight) [ 11]. Motivational interviewing (MI) has recently been shown to offer significant increases in weight losses achieved by standard face-to-face behavioral weight management programs [ 13- 17] and thus represents a potent technique for exploration to enhance online behavioral weight management programs.

MI is a client-centered counseling approach to promoting behavior change by exploring personal reasons for engaging in change in a nonjudgmental, supportive yet directive fashion [ 18]. Key elements of MI are reflective listening, objective feedback, eliciting and amplifying expressions of willingness to change, affirming confidence in one’s ability to make changes, and supporting perceived importance for making changes. This paper offers a detailed description of an MI intervention implemented online within the context of a group-based behavioral weight loss program delivered totally online. To our knowledge, this is the first description of a therapist-delivered individual online MI treatment for weight management.

## Methods

### Study Overview

The MI intervention described here is delivered within the context of a randomized controlled trial (NCT01232699) examining the efficacy of an 18-month long, group-based online behavioral weight control program plus individually administered online MI chat (ie, text-based) sessions relative to the group-based program alone. In total, 398 participants (89.7% (357/398) female, 24.1% (96/398) African American, mean age: 48.4±10.1 years, mean BMI 36.6±6.0 kg/m2) were randomized to the 2 conditions. Participants indicated availability for predetermined meeting times; these intact groups were stratified by their baseline BMI percentile value and then were randomized using a biased coin approach.

**Figure 1:**
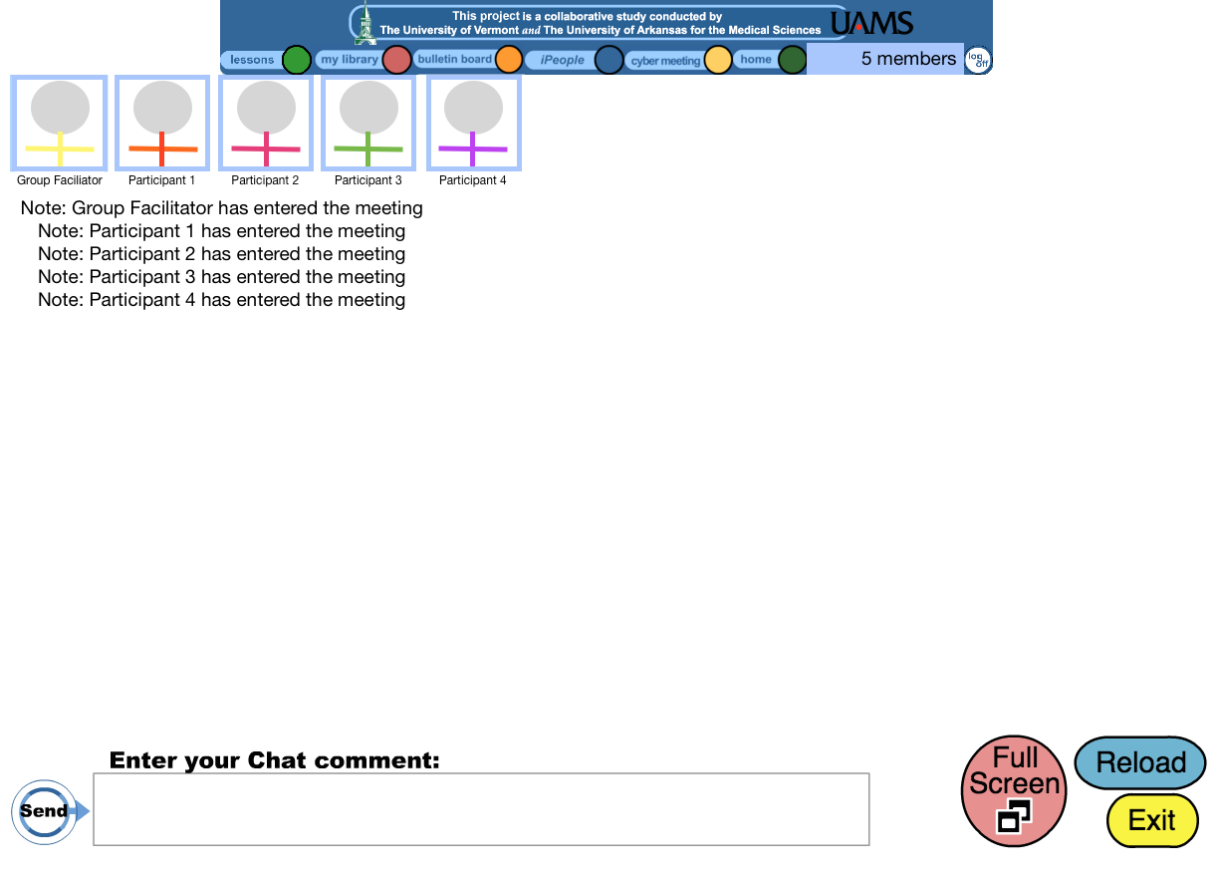
A screenshot of the group chat room utilized in the program.

The online group program utilizes an evidence-based program that achieved an average of 5.5% weight loss in previous research [ 11] and extends this earlier study to determine whether the addition of MI enhances weight losses over and above the evidence-based group program alone. Briefly, the group program is a manualized comprehensive behavioral weight loss program that features 1-hour-long synchronous group chats weekly for 6 months and then monthly for 12 months. The group chat room can be seen in Figure 1where, in order to illustrate the format of the chat room while preserving participant confidentiality, stick figures and pseudonyms (eg, participant 1) are used instead of participant/facilitator photos and names. The group program focuses on the modification of eating and exercise habits by using behavioral strategies (eg, dietary, weight, and physical activity self-monitoring, stimulus control, problem solving, goal setting, relapse prevention, and assertiveness training) and self-management skills (eg, calorie and fat goals, graded exercise goals). The password-protected website includes written lesson materials for each session topic; the lessons include opportunities for the participants to apply the topic of the lesson as “homework.” The website also includes an online self-monitoring tool, a bulletin board for participants to post comments or questions for one another, and helpful hints (eg, recipes, weight loss tips, and updates on local physical activity opportunities). This online group program has been previously described in detail [ 11, 19]; therefore, this paper focuses on the novel online MI intervention, rather than the full study protocol.

The current report is intended to offer an overview of the MI intervention protocol and of the empirical foundation that guided its development. The MI intervention methods described are based on evidence, where available, and on clinical judgment, in the absence of such data. The study is approved by the Committee on Human Research in the Behavioral Sciences at the University of Vermont and the Institutional Review Board at the University of Arkansas for Medical Sciences.

### Measures

The primary outcomes are change in body weight at 6 and 18 months post randomization, measured in-person by a digital scale. Demographic data are obtained by online questionnaire, and process data (eg, self-monitoring of dietary intake and physical activity, group session attendance, emoticon utilization, program evaluation) are collected throughout intervention delivery. The treatment self-regulation questionnaire [ 20] is used to measure autonomous and controlled motivation.

We will use the Motivational Interviewing Skills Code version 2.1 (MISC) [ 21] both to establish treatment fidelity [ 22] and to examine whether participant language mediates weight loss outcomes [ 23]. The MISC software was developed to rate counselor adherence to MI within a session by coding global dimensions and specific therapist behaviors. It has been widely used in a range of research settings [ 24] and offers the advantage of allowing examination of client language as a predictor of treatment outcome [ 23]. This will be crucial because of the modality of the MI sessions; counselors and participants are not able to gauge one another’s nonverbal and auditory cues, such as inflection, tone, or facial expression. In addition, it could be difficult for a participant to determine whether a counselor’s comment is a reflective statement or a question, absent inflection and tone. Similarly, it is unclear whether a participant’s reading of a counselor’s reflective statement or summary will evoke the same response as hearing a counselor’s reflection. It is possible that without nonverbal cues, the written version of a reflection may lack the impact and empathy that a spoken MI reflection or affirmation can provide. For this reason, the intervention emphasizes amplified reflections, double-sided reflections, and summaries, while avoiding simple reflections that could be perceived as very redundant. Although there are several potential disadvantages to a text-based MI interaction, we have the advantage that all MI sessions will have a transcript generated from the chat, which will facilitate these analyses.

### Analysis Plan

First, we will examine the impact of the addition of individual MI counseling to the group-based online weight control program on weight loss (ie, the main outcome), dietary and physical activity self-monitoring, and group session attendance. We hypothesize that the participants who receive MI sessions will self-monitor their diet and physical activity more frequently and attend more group chat sessions, which will lead to greater weight loss among these participants. Second, we will examine the uptake of MI sessions, autonomous and controlled motivation, and qualitative and quantitative evaluation of the MI sessions by participants to inform future delivery of MI online. Previous research [ 25- 27] has reported favorable responses of participants to online motivational interventions for increasing physical activity, although MI in these settings was delivered in a largely automated intervention. The current study will also allow us to explore participant characteristics that may be associated with engagement in the MI sessions. For example, some preliminary evidence suggests that African Americans may find MI less helpful than Caucasian Americans [ 17, 28]. Finally, we will examine utilization of particular features of these MI chat sessions (eg, emoticons, importance/confidence ruler).

### Motivational Interviewing Intervention

Participants randomized to the MI condition receive 6 MI sessions at regularly scheduled intervals throughout the 18-month program. Participants are assigned to an MI counselor separate from their group weight loss facilitator; the assignment is made when participants select a convenient appointment time for their first MI session. Prior to this, the MI counselors post available appointment times on the online scheduler and then send an email inviting those randomized to the MI condition to schedule their first MI chat. The MI counselor posting the time slot that the participant selects then becomes her MI counselor for the duration of the program, in order to foster rapport and permit continuity. (Note: We decided to use feminine pronouns throughout this article to refer to our participants, as the majority of participants in this study are female, as is the case in most weight loss programs [ 29].) To facilitate trust, all conversations between the participant and the MI counselor are kept private, with no information shared with the group facilitator, and participants are aware of this separation from the onset of the MI chats.

The MI sessions are conducted using an interactive, synchronous format of a private chat, integrated within the same website as the weight loss program website. We decided to use text-based chat because many of our study participants live in rural areas without consistent access to high speed Internet, which is required for other types of chat sessions (eg, video chat). Individual MI sessions are conducted, as there is greater evidence supporting 1-on-1 MI compared to group MI sessions for behavior change [ 18]. The MI chat sessions are designed to last approximately 30 minutes and follow a semistructured interview format that allows tailoring of the session to participant concerns and issues, as well as flexibility in the sequencing of topics within the session, while standardizing the range of topics and issues covered across participants. MI chat sessions do not introduce new information about weight loss methods or dietary/physical activity strategies, and specific questions raised by participants related to behavioral strategies or diet or exercise are directed back to the group facilitator. The goal of the MI sessions is to clarify and amplify personal reasons to make behavior changes that promote weight loss and sustained weight maintenance.

### Timing of the Motivational Interviewing Sessions

There are no empirical data examining different patterns of MI delivery within weight management to guide selection of the timeframe for the individual MI sessions. Specifically, there are no data to inform the question of whether it is most effective to front-load the MI sessions and capitalize on the motivational forces that compelled an individual to seek treatment, increasing and extending their personal reasons to engage in weight loss efforts to strengthen their motivation and resolve. Alternatively, one can consider more of a “rescue” approach, which seeks not so much to build on the strength of initial motivation but offer augmentation later in the program when motivation can be flagging. A “middle ground” approach that offers some MI sessions up-front and some as the weight control program unfolds was selected to provide intervention at both of these important phases of the treatment experience. Further, this approach to distribute MI sessions across the treatment program is consistent with the approach successfully used in a previous study that offered individual MI sessions as an adjunct to group weight management but delivered the MI sessions face-to-face [ 14]. The choice of 6 sessions for the current study replicates the number of sessions offered in this previous study.

The first MI session is scheduled after randomization and group assignment ( Table 1), but before beginning the group sessions. Initiation of MI prior to starting the group program allows assessment of the primary motivators for enrolling in the weight loss program and expectations surrounding behavior changes to promote weight loss before any contact with the program psychoeducational materials or the group leader, and thus allows focus on the personal motivators that brought the participants into the treatment program.

**Table 1:** Motivational interviewing session timing, content, and motivational focus.

Session	Timing	Content	Motivational Focus
1	After randomization and before group sessions start	Elicit primary motivators for seeking weight loss treatment, expected outcomes from weight loss, and behavior changes the participant is considering	Evoke change talk focused on selected self-monitoring strategy
2	Week 5 of group program (after 5th group session)	Review objective data on the program engagement (ie, attendance at group chat sessions, submission of self-monitoring records) and perceptions about progress toward goals	Evoke change talk focused on weight control strategies that have been helpful in making progress toward goals and strategies that the participant would like to add to improve weight loss
3	Week 18 of group program (6 weeks before transition to monthly group sessions)	Take stock of progress while considering the good and not-so-good aspects of moving to less frequent meetings; explore whether/how motivators for weight loss have shifted and any plans for maximizing remaining weekly group sessions	Evoke change talk surrounding behavioral goals to maximize desired progress prior to transition to monthly group sessions
4	Week 31 of group program (6 weeks after the transition to monthly sessions)	Reflect on the impact of transition on weight management behaviors, by identifying self-management successes and challenges during this phase and refocusing, if necessary	Evoke change talk about weight control strategies using and/or considering to get back on track toward personal goals
5	Week 44 of group program	Address weight maintenance by focusing on strategies used or considering to maintain commitment to goals and/or to lifestyle behaviors, with focus on the long term after program structure ends	Evoke change talk regarding strategies for sustaining weight loss, re-engaging in weight loss efforts, and/or to reverse weight regain
6	Week 57 of group program	Review accomplishments of which the participant is most proud and identify goals for the final months of program	Evoke change talk regarding strategies for weight maintenance

The second MI session is scheduled after the fifth weekly group session. This point in the treatment program was selected because emerging evidence from our research group indicates that engagement in the initial 4 weeks of a behavioral weight control intervention is predictive of longer-term success [ 19]. Those who self-monitor relevant weight loss behaviors during this first month of treatment achieve greater weight losses than those who are less engaged. Work of others echoes and extends this emphasis on early engagement [ 30, 31], with weight losses in the first or second month significantly associated with long-term weight losses. Collectively, this research dramatically reinforces the importance of getting involved and engaged in the treatment process during the first few weeks of the weight control program and served as the impetus to include an MI session at this influential juncture.

The transition to a less frequent intervention contact schedule is another point that may be critical in the treatment process. Typically, behavioral weight management programs start with weekly group contact and shift to biweekly or monthly groups after 4 to 6 months, and studies suggest that weight loss can be attenuated when group meetings become less frequent, although this is an understudied area [ 32]. Further, clinically, participants can express anxiety or apprehension about leaving the structure of the weekly accountability provided by the group sessions; therefore, in hopes of buffering the impact of the transition from weekly to monthly group meetings at 6 months, the third MI session is scheduled approximately 6 weeks before that change in hopes of offering a motivational boost entering into the transition. The fourth MI session is scheduled approximately 6 weeks after the transition (ie, week 31) to allow the participant to reflect on the experiences associated with the transition and the impact that it has had on their engagement in weight management behaviors and outcomes, offering an opportunity for refocusing if necessary. The fifth and sixth MI sessions are offered at weeks 44 and 57, respectively, when there is time to make some changes before the end of the program, to re-engage in weight loss efforts, and/or to reverse weight regain that might have occurred, should the participant elect to focus on behavior change. These MI sessions are designed to prompt self-reflection on current goals and motivations in contrast with those expressed when starting the program. The goal of these 2 final MI sessions is to facilitate increased self-efficacy and autonomous motivation [ 33] for behavior change and/or to revitalize motivation and purpose, if that is necessary and desired.

### Content of Motivational Interviewing Chat Sessions

All of the sessions use a semistructured interview format that guides the content and suggests the session flow. The content and motivational focus for each session is detailed in Table 1. Each session also provides an opportunity for the participant to add items to the agenda for discussion. In this way, standardization across participants can be balanced with personalization of the sessions based on individual needs and issues.

The first MI session focuses on the participant’s primary motivators for seeking weight loss treatment, expected outcomes from weight loss, and the behavior changes the participant considers most efficacious for her personal weight loss efforts. Specifically, the counselor queries the participant about the reasons that led her to enroll in a weight control program at this point in her life and the behaviors that she is considering changing to achieve weight loss. The counselor also explores with the participant how she thinks her life will be different if she is as successful as possible with her planned behavior changes. With permission from the participant, the counselor provides information on the strong associations between self-monitoring of dietary intake and physical activity early in the program (ie, in the first 4 weeks) and weight loss success. A collaborative discussion about how self-monitoring might figure in the participant’s thoughts about her own personal engagement in the program follows. Among those participants who express interest in using self-monitoring to maximize her personal success, a discussion about the self-monitoring approach (eg, logging foods before consuming them, logging foods at the end of the day) she thinks would be most effective for her is then initiated. The MI counselor utilizes the importance/confidence rulers (with amplification) to evoke change talk regarding the self-monitoring strategy identified. Change talk is at the heart of MI counseling and refers to a participant’s stated desire, ability, reason, and/or need to change behavior, as well as her commitment to changing [ 18]. As in all subsequent MI sessions, the initial session ends with a summary of what has been discussed during the chat followed by a query about the completeness of the summary and a request for the participant to provide annotations of any missing critical elements.

The second MI session includes objective data on the participant’s engagement thus far in the program (ie, her attendance at group chat sessions, her submission of self-monitoring records—not the content of the records or her reported calorie levels but the act of completing the records). In addition, the counselor asks about the participant’s perceptions of her progress toward her goals and the role, if any, that thinking about her primary motivators for weight loss has played in her behavior change efforts thus far. Because the nature of the queries in this MI session can be quite different depending on how the participant is feeling about her progress thus far (ie, is she pleased/excited vs disappointed/frustrated?), the counselors have 2 versions of the semistructured interview to allow tailoring to participant’s self-evaluation; the counselor selects the semistructured interview with the more positive or the more negative self-evaluation based on the participant’s response to the initial question querying about progress thus far. Notably, the initial question does not specify progress about what (ie, progress with weight loss, performance of behavior changes, or any other concrete outcome) but instead offers an open-ended question that allows the participant to focus on whatever dimension she finds most relevant in her self-evaluation.

The third MI session allows the participant a chance to take stock of her progress once again and make plans for how she wants to proceed as she moves into the transition period and has fewer group sessions each month. The format of the session offers deliberation of the good things about transitioning to less frequent meetings and the less-good things to allow participants to explore both aspects of the upcoming change. The content of this session is focused on exploring whether/how the motivators for weight loss have shifted, her perspective on her engagement and progress thus far, and eliciting emotional reactions to the prospect of transitioning to monthly sessions (eg, concerned, excited). Exploration of the steps that she is considering taking to maximize her satisfaction with her progress is also part of this chat. The counselor then utilizes the importance/confidence rulers (with amplification) to evoke change talk regarding the steps that the participant identifies as likely to maximize her satisfaction, with an ultimate goal of negotiating a plan of action to make the most of the next few weeks before transitioning to monthly group sessions.

The fourth MI session highlights current status after transitioning to monthly sessions, identifying any successes in self-management during this phase of the program and exploring any challenges or obstacles encountered. Such discussions foster autonomous motivation, build self-efficacy [ 34], and allow these strengths to be balanced with identification of the changes (if any) that the participant would like to see in her performance and/or her goals for this next phase of the program. Again, the emphasis is on eliciting change talk about weight maintenance related behaviors or about weight loss promoting behaviors. With permission, the counselor provides information from the National Weight Control Registry [ 35] about strategies that other individuals have used to successfully maintain their weight. After providing this information, the counselor elicits participant self-report about strategies she might already be using and which, if any, she might be considering implementing in the near future. The counselor then utilizes the importance/confidence rulers (with amplification) to evoke change talk regarding these strategies.

The fifth MI session centers on strategies that the participant has used or would like to use to maintain her commitment to her goals and to lifestyle behaviors over the long-term, as she will not have the structure of the group chats and other components of the weight control program to support her in these efforts after the end of the program. With permission, the counselor also uses a menu of options to provide information about strategies that others have found helpful in sustaining weight loss [ 36, 37] and elicits reactions to which strategies appeal to the participant.

The final MI session emphasizes the accomplishments the participant is most proud of over the course of her participation in the program, prompting her to review her experiences over the full program to facilitate a balanced perspective of successes and challenges. This conversation also aims to elicit envisioning about the long-term goals, then moves on to discuss how the final 3 months in the program can support this longer-range vision.

The semistructured interview formats provide a template that will be useful in replicating and disseminating the approach taken to deliver the counseling, should the MI sessions prove effective in enhancing weight loss outcomes. Furthermore, the chat format generates a transcript that is used for constructing clinical notes for the counselor and for supervision.

### Motivational Interviewing Techniques and Strategies Employed in Chat Sessions

**Figure 2:**
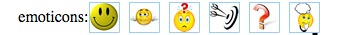
The emoticons used in the motivational interviewing weight loss intervention.

In addition to the specific content areas described above, there are session components that speak to how the MI spirit is embodied in this intervention by the counselor. Present in each chat is an emphasis on collaborative agenda setting and participant-driven goal setting, which is facilitated by counselors who provide a menu of options if participants do not immediately identify their own goals. In addition, open-ended questions are used, with the counselor attempting to maintain a 2-to-1 ratio of reflective statements to questions. The underlying goal for each chat is to foster a strong collaborative relationship in an environment of empathy and respect with an emphasis on evoking the participant’s personal reasons for change and seeking to elaborate and strengthen these reasons. Counselor summaries at the end of the session followed by an invitation to the participant to amend and/or correct as needed are also incorporated into each MI session. Furthermore, counselors use importance and confidence rulers as tools to elicit change talk and fortify self-efficacy, respectively. To facilitate the use of the rulers, a visual representation of a yardstick is graphically displayed in the chat room, and the participant clicks on the location on the ruler which corresponds to where she sees herself. This value is immediately displayed in the chat log (eg, importance=10) to allow the counselor to comment and to guide continued conversation. Finally, in recognition of the lack of nonverbal communication with the online chat format, and the concern that it might be difficult to place text-only comments in an emotional context without voice inflection, we created 6 emoticons that are displayed in the chat room. The 6 emoticons are intended to convey facial expressions or body language that could be seen if the session were face-to-face. Upon selecting an emoticon, the participant’s selection is transformed into text for her counselor to see in the chat log (eg, when she selects the smiley face, the counselor sees the text “I’m smiling” in the chat log). The 6 emoticons (and the text equivalent in parentheses) are: (1) a smiley face (“I’m smiling.”); (2) a smiling person shrugging (“That’s in the ball park but not quite what I mean.”); (3) a smiley face with a question mark (“I’m not certain what you’re saying. Can you tell me more?”); (4) a bull’s-eye with an arrow (“You’re on the right track. Keep going.”); (5) a question mark (“I have a question.”); and (6) a man with bubbles over his head (“I’m giving this some thought.”) ( Figure 2). The behavioral change techniques [ 38] used in the MI sessions are detailed in Table 2.

**Table 2:** Behavior change techniques included in motivational interviewing sessions.

Category	Technique
Goals and planning	Goal setting (behavior)
	Goal setting (outcome)
	Review behavior goal(s)
	Discrepancy between current behavior(s) and goal(s)
	Review outcome goal(s)
	
Feedback and monitoring	Feedback on behavior
	Self-monitoring of behavior
	
Comparison of outcomes	Credible source
	Comparative imagining of future outcomes
	
Self-belief	Focus on past success
	

### Scheduling Motivational Interviewing Sessions

Participants are prompted by email to schedule their MI chat session at the protocol-specified time in the program. An online scheduling system allows participants to schedule the individual chat with her MI counselor at a time that is convenient for her. There is a 2-week window around the protocol-specified week in which the MI session is to occur and during which participants can schedule. An additional week or “grace period” can be added to accommodate rescheduling due to illness, schedule conflicts, etc. If the window closes without an MI session being completed, the participant must wait to chat with her MI counselor until the next window opens. Within each MI chat window, a series of 3 reminder emails are sent (later emails are not sent if the MI session is completed). The first email is sent at the beginning of the 2-week window, the second email is sent 5 business days after the first email, and the third email is sent 7 business days after the first email. We chose to send 3 emails to schedule the MI sessions because we felt that this approach provides an appropriate number of reminders to schedule the session without alienating participants who are not interested in scheduling a session at that point in time.

### The Motivational Interviewing Chat Room

**Figure 3:**
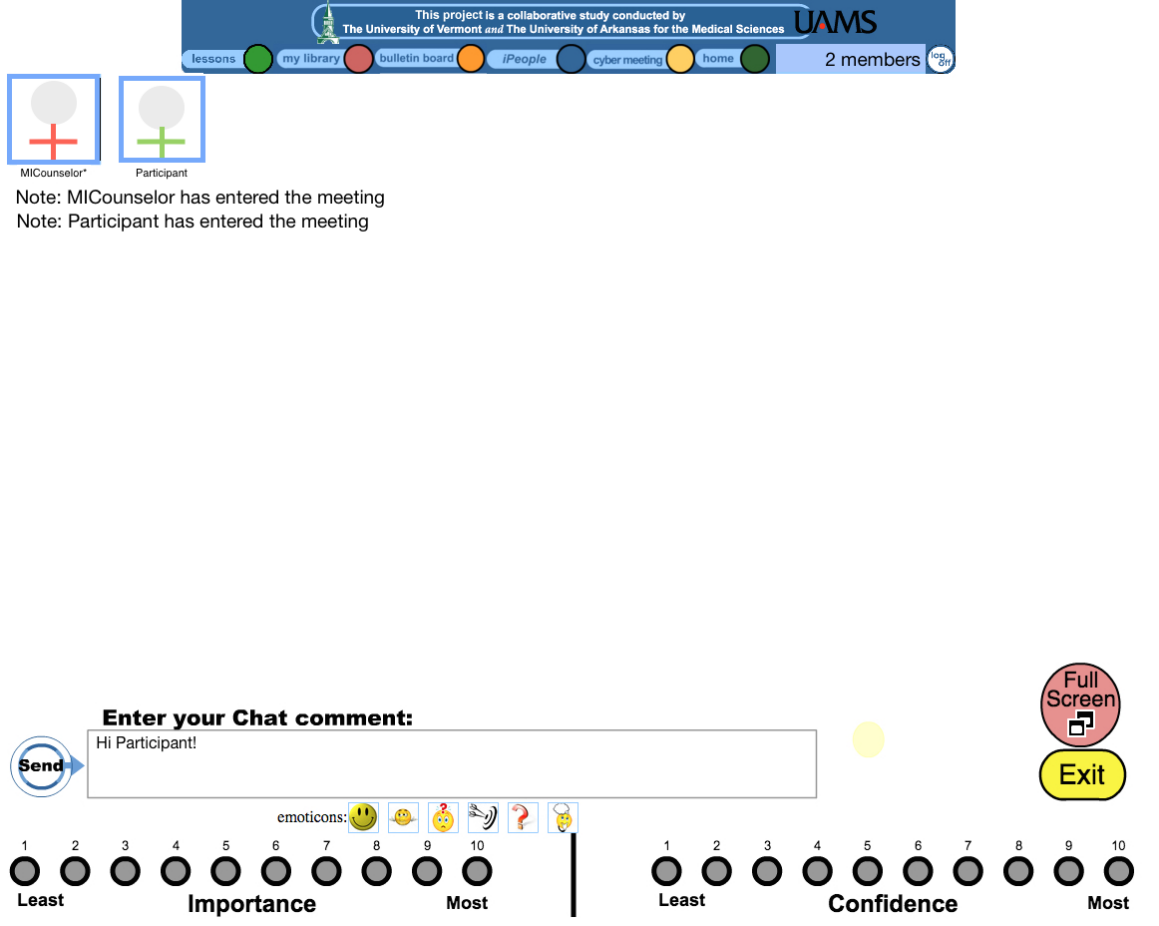
Screenshot of the chat room utilized in the motivational interviewing sessions.

All MI chats are conducted in a private chat room with only the participant and the MI counselor able to access this chat room. This ensures that the conversations between MI counselor and a particular participant remain private and confidential. When the MI counselor or the participant “enters” the chat room, her photograph and her name (or a profile substitute if the participant prefers that over a photograph) appears at the top of the screen. These photographs allow the participant and counselor to “see” each other and to establish that no one else entered the chat room. When someone logs off, or experiences technical difficulties, the photograph and name disappears, which alerts the remaining person of a departure from the chat room. If someone is currently typing text, an asterisk appears next to their name. A screenshot of the MI chat room is shown in Figure 3. Multiple MI chats conducted by separate counselors can occur simultaneously on the study website, but they all occur in separate, private chat rooms. Participants are very familiar with the mechanics of this chatting process as it is the same process we use for the group chats during which the behavioral weight control group program is delivered.

The website was created in Adobe ColdFusion and is run using ColdFusion Server software. The chat room was created using Adobe Flash. The website and the chat room were intentionally created using technologies with minimal participant requirements to facilitate program implementation and eventual dissemination.

### Motivational Interviewing Counselors

The counselors are 5 PhD-level clinical psychologists with previous training and experience with motivational interviewing in face-to-face counseling. Initial training for this study included 20 hours of didactic review of the core tenets of MI and the approach to MI within weight control, and counselors had required readings focused on distinguishing MI from cognitive behavioral treatment within the context of weight management [ 39] as well as the most recent thinking on delivery of MI [ 18]. The senior author (DSW) attended a Motivational Interviewing Network-sponsored training conducted by Dr. William Miller and Dr. Theresa Moyer that focused on training-the-trainer using strategies from the third edition of *Motivational Interviewing*[ 40]. DSW implemented these exercises with the study’s MI counselors. Counselors were required to “test chat” to criterion before being certified to conduct chats with study participants, ensuring that they were comfortable with the features of the chat room and with implementing the semistructured interview format. Online delivery of the MI intervention allows counselors to be geographically distributed, and, indeed, the counselors are located across the country.

Ongoing training and supervision to monitor treatment fidelity and to resolve clinical challenges by consensus is used to enhance standardization. The counselors participate in biweekly supervision-focused conference calls, which identify difficulties in implementing the protocol that may need to be resolved and provide opportunities to problem solve challenging situations. In this ongoing training, we “role-play” by way of reviewing chat scripts to offer opportunities for modeling and for refining protocol scripts about how to handle specific clinical situations that arise. Supervision calls also focus on strategies for increasing attendance at the MI sessions.

## Results

The project was funded in 2010 and enrollment was completed in 2012. Data analysis is currently under way and the first results are expected in 2016.

## Discussion

This paper describes the design of a synchronous, online, 1-on-1 MI intervention currently being tested as an adjunct to an online, group behavioral weight control program to determine whether the addition of the MI chats augment weight loss outcomes compared with the group program alone. MI counseling has typically been delivered face-to-face or by telephone, but there is a growing body of literature examining online strategies to deliver MI-based interventions that target a range of behaviors, including smoking [ 41], substance abuse [ 42- 46], physical activity [ 25, 26], and disordered eating [ 47].

These online interventions are most often delivered via an automated Web-based platform in which elements of a standardized motivational intervention are tailored to a particular participant based on individual characteristics or responses to questions [ 25, 26, 42, 43, 45]. It is clear to participants in these interventions that they are interacting with a computer rather than an actual counselor. In contrast, a few interventions, including the current intervention, provide a synchronous interaction with an actual counselor [ 41, 48]. Intervention with a counselor should allow for greater responsiveness, more accurate but not reiterating reflections, and more nuanced summaries than possible with an automated approach, however sophisticated it might be. This, in turn, might be hypothesized to have greater influence on behavior change. The earlier studies that delivered MI by online counselor personified as an avatar in a virtual chat room [ 41] and in a group format [ 48] have not demonstrated markedly greater effect than the automated-algorithm driven approaches, but this may not be surprising. Group MI has not been shown as robust as individual MI counseling [ 18] and the use of an avatar may have obscured attributions to the actual counselor.

The current study is the first of which we are aware to provide online, synchronous MI counseling delivered by a clinician (without an avatar) to individuals. We had considered conducting the MI sessions by video chat (eg, using video cameras) to emphasize the presence of the counselor and to more closely mirror the face-to-face MI counseling, which has been shown to be effective. However, many of our study participants live in rural areas without consistent access to high speed Internet and have other technical limitations that might preclude their participation in this format of chatting. Therefore, the substantive limits to generalizability that the lack of consistent high-tech access would impose caused us to elect not to use this approach in this study. Use of Internet-delivered weight management treatment is particularly attractive for rural settings that have high rates of obesity and face geographic challenges in access to care [ 36]. However, one of the challenges of text-only chatting is that it is difficult to determine how focused the participant is on the MI session during the counseling time. Many people have the habit of multitasking while on the computer, and when attention is divided by multitasking or interruptions, participants may be less immersed in the MI counseling experience and find it less compelling. This is not so problematic with face-to-face or phone-based sessions, during which divided attention may be more apparent. With the increasing proliferation of FaceTime and other visual chatting platforms that may be more broadly accessible, it may be beneficial in the future to examine the efficacy of video-enhanced MI sessions as this would enable counselors and participants to interpret and respond to subtle changes in inflection, tone, and meaning.

### Conclusions

The current study will add to the body of evidence regarding the efficacy of MI for weight management [ 13- 17], as well as the growing literature on the use of MI in eHealth and mHealth environments [ 25- 27, 41- 45, 47, 48]. Should synchronous online individual MI counseling confer benefits to overall weight loss, there are a range of potential applied benefits that could be readily realized. Many commercial weight loss programs have an online presence for which real-time online MI sessions could augment weight loss outcomes. Further, other group-based lifestyle interventions, whether delivered face-to-face or online, might similarly find it useful to consider an online MI element to enhance their behavior change outcomes.

## Abbreviations

MI: motivational interviewing
MISC: Motivational Interviewing Skills Code software

## References

[1] Ogden CL, Carroll MD, Kit BK, Flegal KM (2014). Prevalence of childhood and adult obesity in the United States, 2011-2012. JAMA.

[2] Knowler WC, Barrett-Connor E, Fowler SE, Hamman RF, Lachin JM, Walker EA, Nathan DM, Diabetes Prevention Program Research Group (2002). Reduction in the incidence of type 2 diabetes with lifestyle intervention or metformin. N Engl J Med.

[3] Pi-Sunyer X, Blackburn G, Brancati FL, Bray GA, Bright R, Clark JM, Curtis JM, Espeland MA, Foreyt JP, Graves K, Haffner SM, Harrison B, Hill JO, Horton ES, Jakicic J, Jeffery RW, Johnson KC, Kahn S, Kelley DE, Kitabchi AE, Knowler WC, Lewis CE, Maschak-Carey BJ, Montgomery B, Nathan DM, Patricio J, Peters A, Redmon JB, Reeves RS, Ryan DH, Safford M, Van DB, Wadden TA, Wagenknecht L, Wesche-Thobaben J, Wing RR, Yanovski SZ, Look AHEAD Research Group (2007). Reduction in weight and cardiovascular disease risk factors in individuals with type 2 diabetes: one-year results of the look AHEAD trial. Diabetes Care.

[4] Blackburn G (1995). Effect of degree of weight loss on health benefits. Obes Res.

[5] Gold BC, Burke S, Pintauro S, Buzzell P, Harvey-Berino J (2007). Weight loss on the web: A pilot study comparing a structured behavioral intervention to a commercial program. Obesity (Silver Spring).

[6] Micco N, Gold B, Buzzell P, Leonard H, Pintauro S, Harvey-Berino J (2007). Minimal in-person support as an adjunct to internet obesity treatment. Ann Behav Med.

[7] Tate DF, Wing RR, Winett RA (2001). Using Internet technology to deliver a behavioral weight loss program. JAMA.

[8] Tate DF, Jackvony EH, Wing RR (2003). Effects of Internet behavioral counseling on weight loss in adults at risk for type 2 diabetes: a randomized trial. JAMA.

[9] Tate DF, Jackvony EH, Wing RR (2006). A randomized trial comparing human e-mail counseling, computer-automated tailored counseling, and no counseling in an Internet weight loss program. Arch Intern Med.

[10] Harvey-Berino J, Pintauro S, Buzzell P, Gold E (2004). Effect of Internet support on the long-term maintenance of weight loss. Obes Res.

[11] Harvey-Berino J, West Delia, Krukowski Rebecca, Prewitt Elaine, VanBiervliet Alan, Ashikaga Takamaru, Skelly Joan (2010). Internet delivered behavioral obesity treatment. Prev Med.

[12] Krukowski RA, Tilford JM, Harvey-Berino J, West DS (2011). Comparing behavioral weight loss modalities: incremental cost-effectiveness of an internet-based versus an in-person condition. Obesity (Silver Spring).

[13] Smith DE, Heckemeyer CM, Kratt PP, Mason DA (1997). Motivational interviewing to improve adherence to a behavioral weight-control program for older obese women with NIDDM. A pilot study. Diabetes Care.

[14] West DS, DiLillo V, Bursac Z, Gore SA, Greene PG (2007). Motivational interviewing improves weight loss in women with type 2 diabetes. Diabetes Care.

[15] Carels RA, Darby L, Cacciapaglia HM, Konrad K, Coit C, Harper J, Kaplar ME, Young K, Baylen CA, Versland A (2007). Using motivational interviewing as a supplement to obesity treatment: a stepped-care approach. Health Psychol.

[16] Rubak S, Sandbaek A, Lauritzen T, Christensen B (2005). Motivational interviewing: a systematic review and meta-analysis. Br J Gen Pract.

[17] Armstrong MJ, Mottershead TA, Ronksley PE, Sigal RJ, Campbell TS, Hemmelgarn BR (2011). Motivational interviewing to improve weight loss in overweight and/or obese patients: a systematic review and meta-analysis of randomized controlled trials. Obes Rev.

[18] Miller W, Rollnick S (2012). Motivational interviewing: Helping people change. 3rd edition, New York, NY: Guilford Press; ISBN.

[19] Krukowski RA, Harvey-Berino J, Bursac Z, Ashikaga T, West DS (2013). Patterns of success: online self-monitoring in a web-based behavioral weight control program. Health Psychol.

[20] Williams GC, Grow VM, Freedman ZR, Ryan RM, Deci EL (1996). Motivational predictors of weight loss and weight-loss maintenance. J Pers Soc Psychol.

[21] Miller W, Moyers T, Ernst D, Amrhein P 1.

[22] Moyers TB, Martin T, Manuel JK, Miller WR, Hendrickson Stacey M L (2005). Assessing competence in the use of motivational interviewing. J Subst Abuse Treat.

[23] Moyers TB, Martin T, Christopher PJ, Houck JM, Tonigan JS, Amrhein PC (2007). Client language as a mediator of motivational interviewing efficacy: where is the evidence?. Alcohol Clin Exp Res.

[24] Madson MB, Campbell TC (2006). Measures of fidelity in motivational enhancement: a systematic review. J Subst Abuse Treat.

[25] Oenema A, Bolman C, Guyaux J, Lechner L, Friederichs Stijn A H, Van Keulen Hilde M (2015). Motivational interviewing in a web-based physical activity intervention: questions and reflections. Health Promot Int.

[26] Friederichs S, Bolman C, Oenema A, Guyaux J, Lechner L (2014). Motivational interviewing in a Web-based physical activity intervention with an avatar: randomized controlled trial. J Med Internet Res.

[27] Moreau M, Gagnon M, Boudreau F (2015). Development of a fully automated, web-based, tailored intervention promoting regular physical activity among insufficiently active adults with type 2 diabetes: integrating the I-change model, self-determination theory, and motivational interviewing components. JMIR Res Protoc.

[28] Befort CA, Nollen N, Ellerbeck EF, Sullivan DK, Thomas JL, Ahluwalia JS (2008). Motivational interviewing fails to improve outcomes of a behavioral weight loss program for obese African American women: a pilot randomized trial. J Behav Med.

[29] Anderson JW, Konz EC, Frederich RC, Wood CL (2001). Long-term weight-loss maintenance: a meta-analysis of US studies. Am J Clin Nutr.

[30] Unick JL, Hogan PE, Neiberg RH, Cheskin LJ, Dutton GR, Evans-Hudnall G, Jeffery R, Kitabchi AE, Nelson JA, Pi-Sunyer FX, West DS, Wing RR, Look AHEAD Research Group (2014). Evaluation of early weight loss thresholds for identifying nonresponders to an intensive lifestyle intervention. Obesity (Silver Spring).

[31] Nackers LM, Ross KM, Perri MG (2010). The association between rate of initial weight loss and long-term success in obesity treatment: does slow and steady win the race?. Int J Behav Med.

[32] Patidar SM, Perri MG, Middleton K M Ross (2012). The impact of extended care on the long-term maintenance of weight loss: a systematic review and meta-analysis. Obes Rev.

[33] Deci E, Ryan R (2008). Self-determination theory: A macrotheory of human motivation, development, and health. Can Psychol.

[34] Markland D, Ryan R, Tobin V, Rollnick S (2005). Motivational interviewing and self-determination theory. J Soc Clin Psychol.

[35] Wing RR, Phelan S (2005). Long-term weight loss maintenance. Am J Clin Nutr.

[36] West DS, Gorin AA, Subak LL, Foster G, Bragg C, Hecht J, Schembri M, Wing RR, Program to Reduce Incontinence by DietExercise (PRIDE) Research Group (2011). A motivation-focused weight loss maintenance program is an effective alternative to a skill-based approach. Int J Obes (Lond).

[37] Wing RR, Tate DF, Gorin AA, Raynor HA, Fava JL (2006). A self-regulation program for maintenance of weight loss. N Engl J Med.

[38] Michie S, Richardson M, Johnston M, Abraham C, Francis J, Hardeman W, Eccles MP, Cane J, Wood CE (2013). The behavior change technique taxonomy (v1) of 93 hierarchically clustered techniques: building an international consensus for the reporting of behavior change interventions. Ann Behav Med.

[39] DiLillo V, Siegfried N, West D (2003). Incorporating motivational interviewing into behavioral obesity treatment. Cogn Behav Pract.

[40] Miller WR, Rollnick S (2012). Motivational Interviewing, Third Edition: Helping People Change (Applications of Motivational Interviewing).

[41] Woodruff SI, Conway TL, Edwards CC, Elliott SP, Crittenden J (2007). Evaluation of an Internet virtual world chat room for adolescent smoking cessation. Addict Behav.

[42] Walters ST, Ondersma SJ, Ingersoll KS, Rodriguez M, Lerch J, Rossheim ME, Taxman FS (2014). MAPIT: development of a web-based intervention targeting substance abuse treatment in the criminal justice system. J Subst Abuse Treat.

[43] Osilla KC, D'Amico EJ, Díaz-Fuentes CM, Lara M, Watkins KE (2012). Multicultural web-based motivational interviewing for clients with a first-time DUI offense. Cultur Divers Ethnic Minor Psychol.

[44] Ondersma SJ, Svikis DS, Schuster CR (2007). Computer-based brief intervention a randomized trial with postpartum women. Am J Prev Med.

[45] Pemberton MR, Williams J, Herman-Stahl M, Calvin SL, Bradshaw MR, Bray RM, Ridenhour JL, Cook R, Hersch RK, Hester RK, Mitchell GM (2011). Evaluation of two web-based alcohol interventions in the U.S. military. J Stud Alcohol Drugs.

[46] Schaub MP, Wenger A, Berg O, Beck T, Stark L, Buehler E, Haug S (2015). A Web-Based Self-Help Intervention With and Without Chat Counseling to Reduce Cannabis Use in Problematic Cannabis Users: Three-Arm Randomized Controlled Trial. J Med Internet Res.

[47] Hötzel K, von BR, Schmidt U, Rieger E, Kosfelder J, Hechler T, Schulte D, Vocks S (2014). An Internet-based program to enhance motivation to change in females with symptoms of an eating disorder: a randomized controlled trial. Psychol Med.

[48] Webber KH, Tate DF, Quintiliani LM (2008). Motivational interviewing in internet groups: a pilot study for weight loss. J Am Diet Assoc.

